# Environmental Stimuli Shape Biofilm Formation and the Virulence of Periodontal Pathogens

**DOI:** 10.3390/ijms140817221

**Published:** 2013-08-20

**Authors:** Marja T. Pöllänen, Annamari Paino, Riikka Ihalin

**Affiliations:** 1 Institute of Dentistry, University of Turku, FI-20014 Turku, Finland; 2 Department of Biochemistry and Food Chemistry, University of Turku, FI-20014 Turku, Finland; E-Mails: aepain@utu.fi (A.P.); riikka.ihalin@utu.fi (R.I.)

**Keywords:** periodontal pathogens, environmental stimuli, biofilm EPS, virulence factors

## Abstract

Periodontitis is a common inflammatory disease affecting the tooth-supporting structures. It is initiated by bacteria growing as a biofilm at the gingival margin, and communication of the biofilms differs in health and disease. The bacterial composition of periodontitis-associated biofilms has been well documented and is under continual investigation. However, the roles of several host response and inflammation driven environmental stimuli on biofilm formation is not well understood. This review article addresses the effects of environmental factors such as pH, temperature, cytokines, hormones, and oxidative stress on periodontal biofilm formation and bacterial virulence.

## 1. Introduction

**Figure 1:**
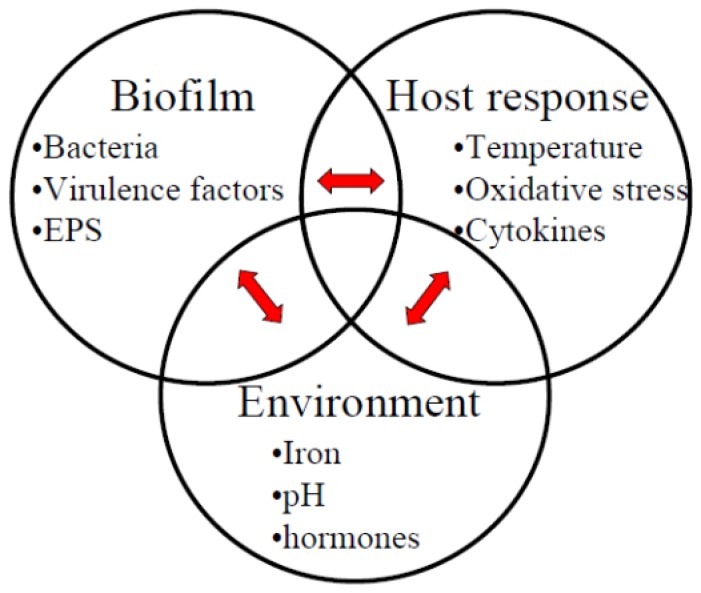
Interactions between periodontal pathogens and host in the subgingival environment.

Periodontitis is a common disease affecting the tooth-supporting structures of millions of people worldwide, and it is a multifactorial disease initiated by bacteria growing as a biofilm at the gingival margin. Periodontal biofilms are diverse, and the nature and communication of these biofilms differs in health and disease. The bacterial composition of periodontitis-associated biofilms has been well documented and is under constant analysis. Several key pathogens have been identified: *Porphyromonas gingivalis*, *Treponema denticola*, *Tannerella forsythia*, *Aggregatibacter actinomycetemcomitans* and more recently also *Filifactor alocis*, *Staphylococcus aureus* and the genus *Desulfobulbus* [1–4]. In addition to bacteria, viruses are commonly detected from periodontal lesions and subgingival plaque samples of patients with aggressive periodontitis [5,6]. Furthermore, the association of the biofilm-forming yeast *Candida albicans* has been detected in approximately 50% of severe chronic periodontitis patients and in only 15% of subgingival samples isolated from healthy patients [7]. Periodontal biofilm formation is a stepwise and continuous process. In the initial phase, the gram-positive and aerobic bacteria dominate. Later, the gram-negative anaerobic periodontopathogens increase in the biofilm. Clinically, the course of periodontitis leads to increased subgingival inflammation and formation of periodontal pockets. The subgingival environment is ideal for the periodontopathogens, being alkaline, hemin and protein rich with various cytokines and hormones. The bacterial cells in biofilm are surrounded by extracellular polymeric substance (EPS), which is composed of polysaccharides, proteins and extracellular DNA, and may account for as much as 90% of the total mass of the biofilm [8–10]. The EPS protects pathogens from host defence cells, such as macrophages, and humoural immune defence factors, such as antibodies and complement, as well as antibiotics. In addition, the high bacterial cell densities in biofilm enable small molecule mediated inter- and intra-species crosstalk, *i.e.*, quorum sensing, which itself may regulate virulence gene expression via pathogens. Moreover, both the host response and the environmental factors in periodontitis can affect the biofilm formation and virulence gene expression of periodontal pathogens (Figure 1). We discuss this crosstalk network of molecular interactions in this review article.

## 2. Host Inflammatory Reaction-Related Stimuli

The oral environment is an ideal, nutrient-rich, warm and growth-promoting place for bacteria to form communities. This first part of the digestive tract offers the bacteria perfect non-shedding surfaces of the teeth to attach and multiply. The bacterial biofilm on the tooth surface activates both the innate and adaptive host responses, which, in turn, have an effect on the biofilm. The first-line host defence is initiated by the polymorphonuclear (PMN) leukocytes that are recruited to the site by chemotactic factors, e.g., the gradient of IL-8 and ICAM-1 in the junctional epithelium [11]. In the subgingival area, the temperature increases, and the inflammatory reaction causes the release of reactive oxygen species (ROS), such as hydrogen peroxide (H_2_O_2_) and superoxide (O_2_^•−^), and cytokines from the host cells to destroy the bacteria. However, the bacterial biofilm is a community, and not only the bacteria but also their virulence and the EPS surrounding them may be affected and changed by the changes produced during the inflammation process.

### 2.1. Temperature

Changes in the environmental temperature have an effect on various functions and virulence of diverse microbial species. Bacteria can sense changes in temperature by proteins, e.g., transcriptional regulators, kinases (e.g., histidine kinase in association with a cytoplasmic response regulator) and chaperones, and via their membrane lipids (for a review see [12]). In the periodontal pocket, an approximately 2 °C increase in the local subgingival temperature has been reported in diseased sites compared with healthy sites [13]. The temperature increase is a host defence mechanism that triggers virulence and heat shock gene expression in bacteria in response [12]. However, the temperature increase also affects the attachment of bacteria, coaggregation of bacteria, and protease production [14–17]. *P. gingivalis* has displayed decreased expression of proteases and down-regulation of the genes coding fimbrial proteins in response to temperature elevation [15,16]. Furthermore, the structure of lipid-A in *P. gingivalis* also appears to be affected by temperature. In elevated temperature environments, increasing amounts of monophosphorylated penta-acylated lipid A are expressed [18]. This lipid A form in turn appears to be a more potent activator of the host Toll-like receptor 4 (TLR4) and further renders the bacteria more susceptible to host defensins [18]. However, the net effect of the temperature increase seems to favour the periodontal pathogens in subgingival biofilms because increased proportions of *Prevotella intermedia*, *P. gingivalis* and *A. actinomycetemcomitans* have been reported in sites with elevated temperatures [19].

### 2.2. Oxidative Stress

ROS generation, as a part of the host defence mechanism or from the initial *Streptococcal* colonisers in the biofilm, induces oxidative stress in other biofilm bacteria and especially in the anaerobic species of the community. The anaerobic bacteria sense the levels of ROS by transcriptional regulators such as OxyR, PerR and OhrR. Superoxide dismutases, alkyl hydroperoxide reductase (AhpC) and catalases are produced by the bacteria for the detoxification of the ROS [20,21]. In the periodontal biofilm community, *F. nucleatum* is important as an intermediate coloniser between the early *Streptococcal* and late anaerobic periodontopathogenic colonisers. *F. nucleatum* appears to facilitate the survival of other anaerobic bacteria in the biofilm [22]. Although an obligate anaerobe, *F. nucleatum* can adapt to oxidative stress in the biofilm and even increase in number in aerobic conditions for up to 2 days [23,24]. The response of *F. nucleatum* to oxidative stress appears to be mediated by the AhpC redox system [25]. Furthermore, the oxidative stress appears to also alter the carbohydrate metabolism of *F. nucleatum* by modifying or increasing the intracellular concentrations of glycolytic enzymes, thereby decreasing ATP production. Increases in the chaperone proteins ClpB and DnaK, the heat shock protein HtpG and the transcription repressor HrcA in *F. nucleatum* in oxidative stress conditions appear to be aimed at diminishing the harmful effects of ROS [25]. In addition, the periodontopathogens have developed strategies to adapt to oxidative stress. For example, *P. gingivalis* produces superoxide dismutase, AhpC, rubrerythrin (rbr), heat shock proteins (HtpG) and chaperons GroEL and DnaK when challenged with oxidative stress [26]. Similarly, elevated inflammatory temperatures upregulate the expression of superoxide dismutase in *P. gingivalis* [27]. Furthermore, the expression of several genes with unknown functions has been observed to be altered in *P. gingivalis* under oxidative stress [28].

### 2.3. Inflammatory Cytokines

In periodontitis, during the active phases of the tissue destruction, the periodontal tissue and gingival fluid typically contain high levels of proinflammatory mediators such as interleukin (IL)-1β [3,29,30]. This overexpression of IL-1β results in host tissue damage, which is characteristic for periodontal disease and aimed at eliminating pathogens together with the regulation of the immune response [31–33]. In periodontium, cytokines are released by first-line human defence cells after identification of periodontopathogens and bacterial products. For instance, gingival epithelial cells and fibroblasts can secrete IL-1β after *P. gingivalis* and *A. actinomycetemcomitans* infection or after being cultured in the presence of *Treponema denticola* lipooligosaccharides [34,35]. Oral bacteria-derived compounds, such as leucotoxins and lipopolysaccharides of *A. actinomycetemcomitans*, induce IL-1β secretion in macrophages [36,37]. Macrophages and monocytes are considered the main producers of IL-1β during inflammation [38–40]. As a key conductor of the inflammatory response, IL-1β induces the release of other proinflammatory mediators [34,41]. Thus, the release of IL-6 and IL-8 from gingival epithelial cells during periodontal infection can be hindered by blocking induction of IL-1β induction [34]. Tumour necrosis factor-alpha (TNF-α), IL-6 and IL-8 are cytokines commonly released by epithelial cells after bacterial infection [42–45]. Recent studies suggest that the gingival cell response differs when infected with oral biofilm [46] or with multispecies biofilms ([47]; reviewed in [48]) as compared with planktonic bacterial infection.

Human IL-1β, crucial for the host battle against pathogens, might also be sensed by host-colonising microbes. The first evidence of bacterial response against cytokines was observed in studies with virulent *Escherichia coli* strains over 20 years ago. It was shown that *E. coli* cells can bind IL-1β and that the growth of these strains increased after IL-1β exposure, whereas treatment with IL-4 or TNF-α was ineffective. Moreover, the use of decoy receptor IL-Ra for IL-1β reversed the growth-promoting effect in *E. coli.* [49] Later, it was demonstrated that other bacterial species such as *Staphylococcus aureus*, *Pseudomonas aeruginosa and Acinetobacter* spp. can alter their growth properties as a consequence of exposure to IL-1β, IL-6 or TNF-α [50].

Gram-positive *S. aureus* has been a central model species for studies of the role of IL-1β in pathogen-cytokine crosstalk. Surprisingly, *S. aureus*, which is most often associated with nasal passages and skin, was also identified from subgingival plaques in 60% of aggressive periodontitis patients belonging to a group of non-smokers [4]. According to an *in vitro* experiment, the growth of *S. aureus* biofilms increases when cultured in the presence of IL-1β [51,52]. The growth enhancement was also observed with two linear peptide fragments (<5 kDa) of human IL-1β [53]. In addition to growth enhancement, the cytokine modulated the gene expression of *S. aureus* biofilms. The cytokine decreased the gene expression of some toxin-encoding genes and increased the expression of host tissue-attachment responsible genes [54].

The hypothesis concerning the capability of the bacteria to specifically bind IL-1β was strengthened by the characterisation of a specific bacterial outer membrane receptor for IL-1β in gram-negative *Yersinia pestis*. The IL-1β-interacting protein is known as a capsule antigen F1 assembly protein (Caf1A), which contributes to capsule antigen (Caf1) transportation across the outer membrane [55]. Interestingly, Caf1 displays 28% sequence homology with human IL-Ra [56]. The second bacterial cytokine receptor, the outer membrane protein OprF, which binds only human interferon-γ, was initially observed in gram-negative *P. aeruginosa.* As a response to cytokine binding, *P. aeruginosa* increased lectin-encoding gene (*lecA)* expression in a quorum sensing dependent manner [57]. The hydrophobic galactose-binding lectin localised in the EPS of *P. aeruginosa* biofilms contributes to species biofilm formation and endothelial cell adherence [58].

Cytokines, such as IL-1α, IL-1β, IL-2, IL-3, IL-4, IL6, IL-7 [59] and TNF [60], use their carbohydrate-binding domains to recognise specific oligosaccharide ligands (reviewed in [61]). The receptor-binding site of IL-1 associates with its cognate receptor, and the second domain, localised opposite to the receptor binding sites, interacts with carbohydrate. For instance, IL-1α and IL-β have different carbohydrate binding activities. Whereas IL-1α binds N-glycan with two α-2–3-linked sialic acid residues, IL-1β recognises the α2–3-sialylated β-galactosyl-ceramides, which have very long and unusual long-chain bases [62]. Moreover, some lipooligosaccharides of *Haemophilus* species are sialylated, and they encode sialyltransferases [63,64].

*A. actinomycetemcomitans* is the only major periodontopathogen to our knowledge that has been shown to both sense and bind IL-1β. The clinical strains of the species display a physiological response to cytokines by decreasing their metabolism and by increasing their biofilm mass [65]. *A. actinomycetemcomitans* biofilms co-cultured with an organotypic gingival mucosa model bind IL-1β, but the use of antibiotics during co-culturing inhibits IL-1β binding [66]. Moreover, the species appears to uptake IL-1β as the cytokine has been detected in the intracellular space of the bacterium [66]. Two intracellular proteins, the trimeric form of ATP synthase subunit β and bacterial histone-like protein HU, have displayed interaction with human IL-β [65,66]. The interaction of internalised IL-1β with a key protein in cellular energy production and genomic DNA condensing HU protein might explain the above-described physiological responses of *A. actinomycetemcomitans.* These results suggest that viable *A. actinomycetemcomitans* cells possess a specific uptake mechanism for IL-1β. According to our recent results, *A. actinomycetemcomitans* encodes a *Pasteurellaceae*-specific outer membrane lipoprotein responsible for IL-1β interaction [67]. In addition to bacterial species, herpesviruses and yeasts found in subgingival biofilms can sense and bind the cytokines produced by the host [68,69].

## 3. Periodontitis-Associated Environmental Factors

Various environmental factors, other than those strictly related to the inflammatory response of the host, may change during the progression of periodontitis. Such factors include, for example, pH, the concentration of iron and hemin, and the presence of various host hormones. All these factors can be sensed by at least some of the periodontal pathogens, although studies at the molecular level are scarce. However, the results obtained thus far suggest that elevated pH and iron limitation may enhance the biofilm formation of some species and influence virulence gene expression, which, in turn, might alter the host inflammatory response.

### 3.1. Alkaline pH

The environment of the periodontitis-associated gingival pocket, and especially the gingival crevicular fluid, is characterised by alkaline pH, which may rise above 8.5 [70–72]. Some periodontal pathogens, such as *P. gingivalis*, *P. intermedia* and *F. nucleatum*, are able to elevate the ambient pH by fermenting amino acids *in vitro* [73], a feature which may also alkalify the microenvironment in subgingival biofilm locally.

An alkaline pH of 8.2 has been shown to increase cell surface hydrophobicity as well as induce the co-adhesion and biofilm formation of *F. nucleatum*, which is accompanied by decreased intracellular polyglucose content and elongation of individual cells [74]. When grown at a slightly lower pH than 8.2, *i.e*., at pH 7.8, *F. nucleatum* cells display upregulated expression of the enzyme formiminotetrahydrofolate cyclodeaminase, which could be involved in raising the surrounding pH value [75]. In addition, the production of a non-iron redox acceptor flavodoxin, the expression of which is typically upregulated in iron-limited growth conditions [76], was also observed to be upregulated in alkaline pH conditions [75]. *F. nucleatum* cells appear to change their metabolism in response to high pH, because some enzymes of the glycolytic pathway, as well as glutamic acid and histidine catabolism, are downregulated in planktonic cells grown at an alkaline pH of 7.8 [75]. However, when the pH is further raised to 8.2, which induces biofilm formation, the amounts of glycolytic enzymes do not increase, whereas glucose storage and lactate production increases [77]. In contrast to increased glucose storage, the production of various proteins involved in protein synthesis decrease at high pH values in *F. nucleatum* biofilm [77]. At pH 8.2, another change in the expression of metabolic enzymes is the increased production of glutamate dehydrogenase, which might indicate that the bacterium adjusts its metabolism to the increased concentration of glutamate in the gingival crevicular fluid associated with inflamed periodontal tissue [77]. The cellular stress response in bacteria might also be activated at an alkaline pH because *F. nucleatum* cells upregulate the expression of peptidyl-prolyl *cis*-trans isomerase (PPI) and the heat-shock protein GroEL [75,77]. Of these proteins, GroEL might have a role in the host-bacterium crosstalk because GroEL-like proteins may modulate the host immune response, as shown with *F. nucleatum* [78] and *A. actinomycetemcomitans* [79]. Moreover, GroEL might be a link between periodontitis and systemic diseases, such as atherosclerosis, as *F. nucleatum* GroEL induces various risk factors of atherosclerosis in mice [80].

Whereas some of the pH-regulated intracellular proteins may alter the virulence of the bacterium, one interesting group of pH-regulated proteins are expressed in the cellular envelope of the bacterial cell. In particular, the proteins that are involved in adhesion to other periodontal pathogens as well as to host cells may play important roles in virulence. When *F. nucleatum* was grown at an alkaline pH of 8.2, elevated levels of FomA adhesion isoforms were detected [77]. FomA may function as recruiter of other periodontal pathogens, such as *P. gingivalis*, [81], and has been proven to be a possible vaccine target in a mouse study [82]. Some of the downregulated cellular envelope proteins of *F. nucleatum* are involved in ATP synthesis and maintenance of a neutral cyto- or periplasmic pH, indicating decreased metabolic activity and adjustment to alkaline conditions, respectively [83]. Some of the downregulated proteins likely have dual roles, as in the case of butyrate-acetoacetate CoA transferase, which is involved both in energy metabolism and, as a virulence factor, butyric acid production [83]. The group of cellular envelope proteins that were upregulated at an alkaline pH contained at least two putative surface antigens: outer membrane protein (OMP), belonging to the Omp IP family of porins, and a pathogen-specific membrane antigen that was predicted to have high affinity to Fe^2+^ [83]. In addition to the Omp IP family porins, the expression levels of various transporter proteins was altered when *F. nucleatum* cells were grown in biofilm at a pH of 8.2, which could be an indication of a changed need to uptake various solutes from the microenvironment in the biofilm [77]. Most of the studies that have investigated the effects of alkaline environment on periodontal pathogens have been performed in *F. nucleatum*. However, up to 50% of the alkaline pH-regulated genes coding *F. nucleatum* cell envelope proteins could have been acquired through horizontal gene transfer, and similar proteins are also found in other periodontal pathogens, such as *P. gingivalis* and *Treponema denticola* [83].

### 3.2. Iron and Hemin

Because free iron catalyses the formation of toxic free radicals from H_2_O_2_ and is essential for the function of both the host and the pathogenic bacteria, human organs have developed complex ways to limit the availability of free iron in the environment [84]. Thus, the environment that surrounds potential colonisers when they enter a human host is suboptimal in terms of free iron concentration. Most of the host iron is bound to iron-binding proteins such as transferrin, ferritin, lactoferrin and haemoglobin that contain hemin or haem. However, the situation may change during the progression of periodontitis. It has been hypothesised that the concentration of hemin may increase due to the increasing concentration of haemoglobin leaking from vascular ulcers of the gingival pocket. Some periodontal pathogens, such as *P. gingivalis*, *T. denticola* and *A. actinomycetemcomitans*, have been demonstrated to express hemin-binding proteins on their surfaces [85–87] that may facilitate iron acquisition in free iron-limited conditions. Moreover, hemin may also directly regulate the virulence characteristics of *P. gingivalis* [88].

Iron chelation has been shown to increase the expression of the EPS-, fimbrial-, and LPS-related genes, *pgaC*, *tadV*, and *rmlB*, respectively, in *A. actinomycetemcomitans*, which also leads to increased biofilm formation [89]. The effect of limited concentrations of iron most likely is mediated by small regulatory RNAs (sRNA) in *A. actinomycetemcomitans*, though the target genes for these sRNAs, have not been yet identified [90]. The most studied periodontal pathogen, regarding the need and effects of iron on the bacterium, is likely *P. gingivalis*. In agreement with being an essential growth factor and important cause of oxidative stress (for review see [9]), iron limitation upregulates the genes involved in iron uptake and downregulates the genes associated with the storage of iron as well as the oxidative stress response of *P. gingivalis* [9]. Moreover, by limiting iron and hemin availability, the host can also increase biofilm formation, the invasion of single bacterial cells to the host cells [9], and increase vesicle secretion and protease production of *P. gingivalis* [91]. Another potential virulence factor of *P. gingivalis* that is regulated by hemin is LPS, and more specifically, the lipid A form of it [88]. A form of lipid A, which is a Toll-like receptor (TLR) 4 antagonist, is produced at high hemin concentrations, whereas at low hemin concentrations TLR4-agonist lipid A is the major form, suggesting that *P. gingivalis* can alter the host response with the changing hemin microenvironment [88].

### 3.3. Hormones

Although various bacterial species are known to respond to the stress-related hormones adrenaline and noradrenaline, and the molecular players of their sensory machinery have been clarified in detailed (reviewed in [92]), the periodontal pathogens have been little studied. In the first study that investigated the effects of catecholamines, noradrenaline and adrenaline, on periodontal bacteria, both negative and positive growth effects on planktonic species were reported [93]. However, both catecholamines inhibited the growth of “red complex” [94] periodontal pathogens *P. gingivalis* and *T. forsythia* (formerly *Bacteroides forsythus*) as well as *A. actinomycetemcomitans* serotypes a and b and *F. nucleatum* [93]. Thus, the authors stressed the importance of negative growth effects and hypothesised that these species might also use catecholamines to enhance virulence gene expression [93]. A recent study by Saito *et al.* [95] demonstrated that growth inhibition of *P. gingivalis* by noradrenaline is accompanied with enhanced production of the virulence-associated protease arg-gingipain B and downregulation of the genes coding polysaccharide biosynthesis-related proteins.

The most significant rise in the levels of the female sex hormones, oestrogen and progesterone, occurs during pregnancy [96]. Although pregnancy gingivitis is currently categorised under the class of “dental plaque-induced gingival diseases modified by the endocrine system” [97], the effects of hormone levels on the progression of gingivitis during pregnancy is still under debate [98–100]. It appears that even though gingival inflammation may intensify during pregnancy, the hormones themselves may cause controversial changes in the periodontium, including decreased inflammatory reactions [101–104] and changes in the composition of subgingival biofilm [105–107]. Moreover, some gram-negative periodontal pathogens, such as *Prevotella melaninogenica*, *P. intermedia* and *P. gingivalis* are able to take up estradiol and progesterone, which the *Prevotella* species may use as a growth factor instead of vitamin K [108]. However, whether these hormones affect the biofilm formation and the virulence of periodontal pathogens is not known.

## 4. Conclusions

In periodontitis, the crosstalk between the host and the bacterial biofilm is diverse and bidirectional. The host response and environmental changes induce stress in the biofilm bacteria (Table 1). Elevated temperature of the subgingival environment, though aimed to eliminate the pathogens appears to only decrease virulence factors in pathogens (e.g., proteases in *P. gingivalis*) and does not sufficiently eliminate the pathogens [19]. The change in the local pH towards an alkaline environment appears to play an important role in the shift towards periodontopathogenic biofilm composition. The biofilm mass is increased in alkaline conditions and, in particular, the intermediate coloniser *F. nucleatum* displays increased adhesion and coaggregation with other bacteria. Oxidative stress and the inflammatory cytokine IL-1β result in decreased metabolism in periodontal biofilm but still increase various virulence factors as well as biofilm formation. The limited amount of free iron appears to enhance biofilm EPS formation [89]. During periodontal inflammation, the increased amount of hemin might downregulate the expression of bacterial virulence factors and upregulate the expression of immune-suppressing molecules. In summary, the environmental changes generated in inflammation favour biofilm formation and appear to drive the bacteria into the shelter provided by the EPS and the lower metabolic activity. The inflammatory environment with active immune cells and a hostile humoural response is not ideal for planktonic bacteria, which are released by mature biofilm when expanding to new habitats. Biofilm formation might explain the onset of less progressive phases in periodontal inflammation and tissue destruction and allow the periodontal pathogens to persist in subgingival spaces.

## Figures and Tables

**Table 1:** Environmental stimuli affecting periodontal biofilm and bacterial virulence factors.

Stimuli	Effect	Species	References
Elevated temperature	Proteases ↓	*Porphyromonas gingivalis*	[16]
	Fimbrial proteins ↓		[15]
	TLR4 activating lipid-A ↑		[18]

Oxidative stress	ATP production ↓	*Fusobacterium nucleatum*	[25]
	Chaperones ClpB, DnaK ↑		[25]
	Heat shock protein HtpG ↑		[25]
	Transcription repressor HrcA ↑		[25]

Oxidative stress	Chaperones ClpB, DnaK ↑	*Porphyromonas gingivalis*	[26]
	Heat shock protein HtpG ↑		[26]
	superoxide dismutase ↑		[26]

Inflammatory cytokine IL-1β	Biofilm formation ↑	*Aggregatibacter actinomycetemcomitans*	[65]
	Metabolism ↓		[65]

Alkaline pH	Co-adhesion ↑	*Fusobacterium nucleatum*	[74]
	Biofilm formation ↑		[74]
	Flavodoxin ↑		[75]
	Glucose storage ↑		[77]
	Lactate production ↑		[77]
	Protein synthesis enzymes ↓		[77]
	Glutamate dehydrogenase ↑		[77]
	PPI and GroEL ↑		[75,77]
	FomA adhesion isoforms ↑		[77]
	ATP synthesis proteins ↓		[83]
	Butyrate-acetoacetate CoA transferase ↓		[83]
	Surface antigens Omp IP ↑		[83]

Iron-limitation	EPS (*pgaC*) ↑	*Aggregatibacter actinomycetemcomitans*	[89]
	Fimbrial (*tadV*) ↑		[89]
	LPS (*rmlB*) ↑		[89]
	Biofilm formation ↑		[89]

Iron limitation	Iron uptake ↑	*Porphyromonas gingivalis*	[9]
	Iron storage ↓		[9]
	Oxidative stress response ↓		[9]
	Biofilm formation ↑		[9]
	Host cell invasion ↑		[9]

High hemin concentration	Proteases ↓	*Porphyromonas gingivalis*	[91]
	Vesicles ↓		[91]
	TLR4 inactivating lipid A ↑		[88]

Noradrenaline	Growth ↓	*Porphyromonas gingivalis*	[93]
	Arg-gingipain B ↑		[95]
